# Blood lead concentrations among pediatric patients with abdominal pain: a prospective cross-sectional study

**DOI:** 10.1186/s12876-021-02023-w

**Published:** 2021-12-20

**Authors:** Amirhossein Hosseini, Anahita Fayaz, Hossein Hassanian-Moghaddam, Nasim Zamani, Seyed Kaveh Hadeiy, Narges Gholami, Naghi Dara, Katayoun Khatami, Pejman Rohani, Scott Phillips

**Affiliations:** 1 Pediatric Gastroenterology, Hepatology and Nutrition Research Center, Research Institute for Children’s Health, Shahid Beheshti University of Medical Sciences, Tehran, Iran; 2 School of Medicine, Shahid Beheshti University of Medical Sciences, Tehran, Iran; 3 Social Determinants of Health Research Center, Shahid Beheshti University of Medical Sciences, Tehran, Iran; 4 Department of Clinical Toxicology, Loghman-Hakim Hospital, Shahid Beheshti University of Medical Sciences, South Karegar Street, Tehran, Iran; 5 Department of Pediatrics, Loghman-Hakim Hospital, Shahid Beheshti University of Medical Sciences, Tehran, Iran; 6 Washington Poison Center, University of Colorado Anchutz Medical Campus, Rocky Mountain Poison & Drug Safety, Denver, CO, Seattle, WA USA

**Keywords:** Lead, Abdominal pain, Pediatric

## Abstract

**Background:**

Lead exposure is one of the most menacing of environmental exposures, particularly in children. Children are more susceptible to the effects of lead which manifest in many organ systems, including interference with mental and motor development. Lead poisoning can cause colicky abdominal pain. In this study, the authors sought to evaluate the prevalence of elevated blood lead level (BLL) and its contributing factors among pediatric patients presenting with abdominal pain. An epidemic of lead poisoning in adults was previously uncovered, and thus a concern for pediatric lead poisoning was raised.

**Methods:**

Pediatric patients presenting to two pediatric clinics in Tehran with abdominal pain were eligible for enrollment in a descriptive prospective cross-sectional study. A predesigned questionnaire was filled for each patient by their consenting parents. The questionnaire queried demographic information, environmental, social, and other relevant parameters for lead exposure. After completion of the questionnaire, biometrics were obtained, and a blood sample was taken from each patient for measurement of BLL and complete blood count.

**Results:**

A total of 187 patients were enrolled in the study. Of them, almost 20% had BLL ≥ 5 µg/dL. Univariate analysis showed that age (*p* = 0.002, OR 3.194, CI 95% 1.504–6.783), weight (*p* = 0.009, OR 2.817, CI 95% 1.266–6.269), height (*p* = 0.003, OR 3.155, CI 95% 1.443–6.899), and playing with both plastic and cotton toys (*p* = 0.03, OR 2.796, CI 95% 1.072–7.295) were significant predictors of high BLLs. Maternal level of education correlated with blood lead concentrations (*p* = 0.048, OR 2.524, CI 95% 1.006–6.331).

**Conclusions:**

A clinically significant number of cases of abdominal pain may have high BLLs. Specific attention should be paid to children presenting with abdominal pain, especially due to the detrimental effects of lead on their mental and motor development.

## Background

Lead poisoning is a common global problem and may present with abdominal pain. Lead is a greyish heavy metal. Because of lead’s physical characteristics, it is vastly used in industry. Lead poisoning is counted to be one of the hazardous environmental exposures. Source for lead poisoning includes paints, water, soil, air pollution, old metal pipes, herbal and traditional medicines, and occupational exposures such as soldering [1].

Lead poisoning is generally asymptomatic making lead screening an important public health issue. However, neural and gastrointestinal (GI) involvement are the most common presentations. Lead poisoning has classically been reported to present with abdominal pain, constipation, nausea, vomiting, lethargy, anorexia, irritability, fatigue, and seizures [2]. It also may cause stupor, ataxia, coma, reduced IQ, anemia, nephropathy, hypertension, and infertility [1]. Although the U.S. Centers for Disease Control and Prevention (CDC) has set a blood concentration of 5 µg/dL as the level necessitating intervention [3], there are pieces of evidence that lead exerts its detrimental effects even in lower levels. Lead poisoning neuro-cognitive and growth impacts are special concerns [4–6]. Clinical studies of pediatric lead poisoning among Iranian children are scarce; however, several sources have been proposed including adulterated opium, soil, rice, water, dust, and air pollution [7–10].

A massive outbreak of lead poisoning happened in Iran in 2016–2017 due to adulterated opium. It is estimated that around 40,000 cases of lead poisoning occurred during this period [8, 9]. Opium smoking with inhalation of lead particles can cause lead poisoning both in the person who smokes it and those who reside in the same place with him. This way, many children were exposed to lead particles and the frequency of lead poisoning increased in the children, as well [11, 12].

During the epidemic of lead poisoning in adults in Iran, the main problem was to diagnose lead poisoning in patients who referred to general emergency rooms throughout the country with non-specific symptoms including abdominal pain. Many of these patients underwent non-necessary diagnostic and therapeutic measures including laparotomy before reaching the final diagnosis of lead poisoning. This could have happened in the pediatric populations, as well. On the other hand, abdominal pain is a rather common symptom in children with different background diseases. This study aims to evaluate the prevalence of lead poisoning in children who refer with abdominal pain to two pediatric academic referral hospitals.

## Methods

Pediatric patients presenting to the GI clinic of Mofid Pediatric Hospital and the pediatric gastroenterology clinic of Loghman-Hakim Hospital with the chief complaint of abdominal pain were eligible for enrollment. Those younger than 18 years of age were considered for enrollment. The goals and objectives of the study were explained to guardians, and if agreed, written consent was obtained. A pre-enrollment standardized questionnaire was provided to the parents. Questions included the patients' age, sex, weight, height, address, duration of living in their current home, age of the residential building the patients and their families resided in, history of recent renovation and painting, type of the wall paint and pipes of their home, history of playing with soil, living near industrial zones (confirmed through municipality map), type of the toys the children played with, parents' level of education and occupation, accompanying signs and symptoms with abdominal pain (e.g. nausea, vomiting, extremity pain), location and quality of the pain, onset and duration of the pain, history of previous visits for abdominal pain and treatments performed, abdominal imaging and endoscopy results and abdominal surgery (if performed), history of treatments for lead poisoning, special diet (if any), using frequent (more than 1–2 times per week) amounts of spices (e.g., turmeric’s color and weight may be augmented with lead) or herbal medicine [13, 14], and presence of ice eating or pica. Findings in the abdominal examination, blood lead level (BLL), and complete blood count (CBC) results were also recorded. Blood samples were then taken, after appropriated skin preparation using ethyl alcohol, to measure BLL and CBC. BLL was measured using the atomic absorption technique and by using Lead care II device (finger prick).

The analysis was performed using statistical package for social sciences (SPSS) software version 24 using Kolmogorov Smirnoff test to find the distribution patterns of quantitative variables, Chi square test to evaluate the differences of qualitative variables and T-test / Mann Whitney U test to test parametric and non-parametric quantitative variables. Cutoff points were determined using the ROC curve test for the variables which showed significant differences among patients with normal and elevated BLL. Multivariate analysis was conducted using binary logistic regression by entering variables with P values less than 0.2 in the model.

## Results

**Fig. 1:**
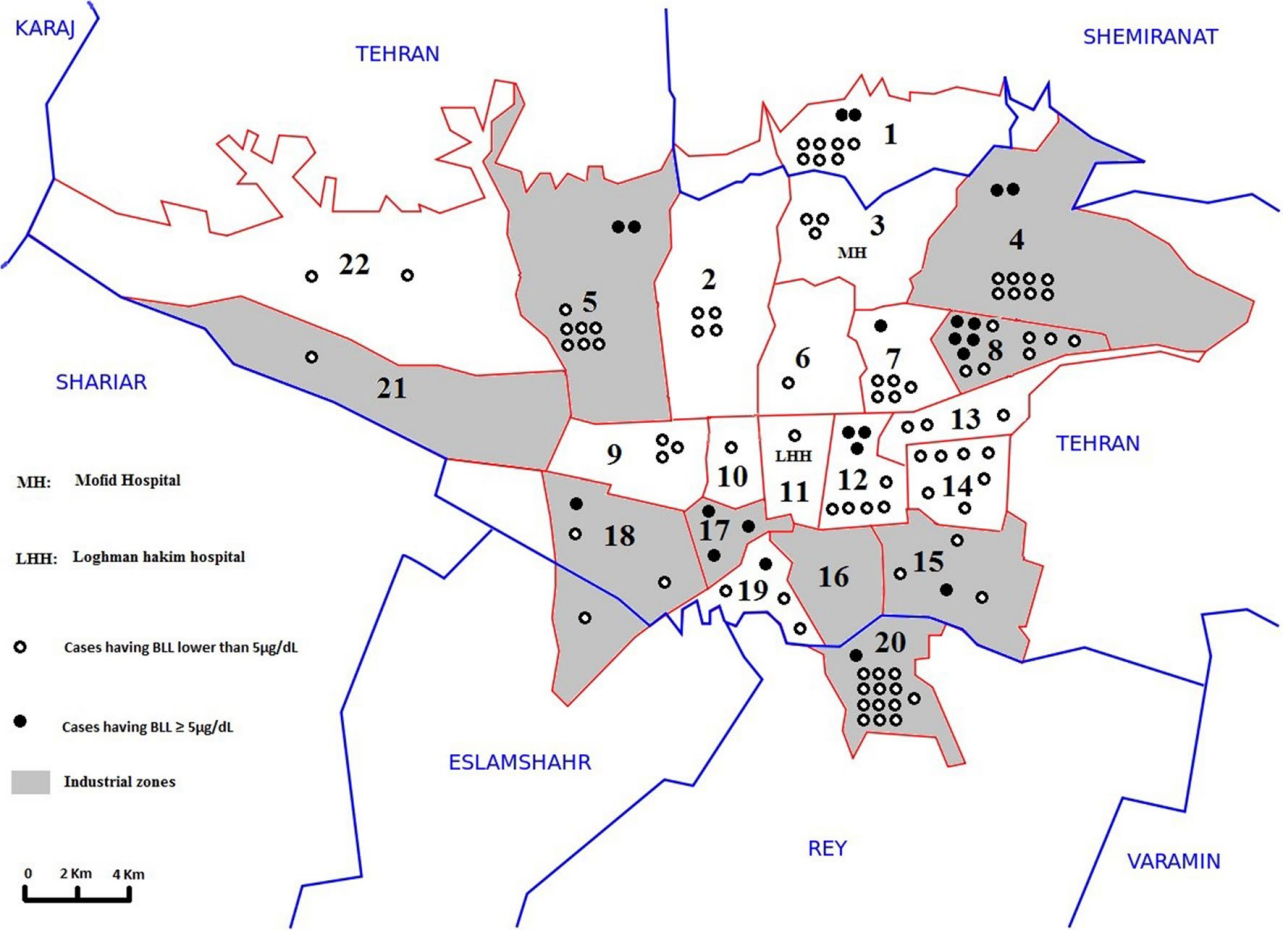
High Lead level distribution in Tehran city (n = 109)

Of the 187 patients who were entered into the study, 101 (54%) were female. The median age was 8 years old [IQR 4, 10] (range;1 to 18). Of them, 165 and 21 were admitted to Mofid and Loghman-Hakim Hospitals, respectively; 109 (58.3%) reported Tehran city as their address and other 78 (41.7%) were either from other provinces or did not answer to the question. Figure 1 shows the distribution of patients referred from Tehran city. The onset of the abdominal pain was acute in 7 (3.8%), chronic in 76 (41.1%), and cyclic in 102 (55.1%) patients. Thirty-six cases (19.3%) had BLLs higher than 5 µg/dL and eight (4.3%) had BLLs over 10 µg/dL. Complete blood count results have been depicted in Table 1.

**Table 1:** CBC results of the patients

	Patients with BLL ≥ 5 µg/dL	Patients with normal BLL	Total	*P* value
*WBC*				
Mean ± SD	8.81 ± 2.58	8.79 ± 2.55	8.79 ± 2.55	0.86
Min–max	4.29–16.8	4.20–17.60	4.20–17.60	
*RBC*				
Mean ± SD	4.17 ± 0.72	4.19 ± 0.82	4.18 ± 0.80	0.24
Min–max	2.80–5.20	2.80–5.90	2.80–5.90	
*HCT*				
Mean ± SD	37.95 ± 5.09	37.16 ± 6.27	37.31 ± 6.06	0.58
Min–max	31.90–54	25–56	25–56	
*Hgb*				
Mean ± SD	11.66 ± 1.14	11.56 ± 1.46	11.58 ± 1.40	0.94
Min–max	9.40–13.90	8.50–15.30	8.50–15.30	
*PLT*				
Mean ± SD	275.40 ± 91.75	292.90 ± 101.72	289.53 ± 99.88	0.36
Min–max	129–454	128–702	128–702	
*MCV*				
Mean ± SD	80.28 ± 6.83	77.62 ± 7.45	78.13 ± 7.39	0.08
Min–max	65–100	54–98	54–100	

Patients with normal BLL (BLL < 5)

In those living in Tehran, districts 7, 12, and 17 had the highest prevalence of elevated BLL (Fig. 1).

Univariate analysis found significant differences in age, weight, height, and use of cotton and plastic toys among patients with normal and elevated BLLs (Table 2).

**Table 2:** Univariate analysis among patients with elevated BLL (n = 187)

Variable	Odd ratio (95% CI)	P value	Variable	Odd ratio (95% CI)	*P* value
**Epidemiological Characteristics (yes vs. no)**			**Symptoms (Yes vs. no)**		
Gender (male vs. female)	1.61 (0.77–3.35)	0.20	Constipation	1.23 (0.54–2.83)	0.62
Age (lower than 5 years old)	3.19 (1.50–6.78)	0.002	Withholding	0.71 (0.07–7.03)	0.77
Weight (≥ 23.5 kg)	0.35 (0.16–0.79)	0.009	Diarrhea	1.08 (0.22–5.25)	0.92
Height (≤ 103.5 Cm)	3.15 (1.44–6.90)	0.003	Nausea	0.60 (0.23–1.55)	0.28
**Accommodation details (Yes vs. no)**			Vomiting	0.60 (0.23–1.55)	0.28
Living in Tehran	0.81 (0.36–1.83)	0.62	Dark stool	0.88 (0.27–2.84)	0.83
Recent renovation of the living place	1.30 (0.55–3.09)	0.55	Dysuria	0.45 (0.13–1.58)	0.20
Living near to industrial zones	0.83 (0.16–4.16)	0.81	No gas passing	1.08 (0.22–5.22)	0.93
Metal pipes used in the living place	1.17 (0.52–2.63)	0.69	Blowing	1.66 (0.54–5.11)	0.37
Wall sleek oil colored	0.47 (0.18–1.21)	0.11	Lack of concentration	0.70 (0.18–2.72)	0.60
Wall opaque oil colored	0.46 (0.10–2.10)	0.30	Difficult child	0.95 (0.10–8.79)	0.97
Plastic wall pain	1.44 (0.59–3.52)	0.42	Learning disability	1.45 (0.17–12.42)	0.73
Multicolor wall paint	1.87 (0.61–5.69)	0.26	Anemia	0.70 (0.14–3.64)	0.67
Wallpaper	0.83 (0.09–7.37)	0.87	Heartburn	0.95 (0.25–3.56)	0.94
**Medical history (yes vs. no)**			Headache	1.12 (0.30–4.14)	0.86
Meconium	0.97 (0.95–0.99)	0.32	Loss of appetite	1.73 (0.62–4.81)	0.29
Soiling	0.99 (0.98–1.01)	0.62	Muscle weakness	0.45 (0.13–1.58)	0.20
Conduction of endoscopy (yes vs. no)	0.43 (0.18–1.06)	0.06	Pain in extremities	0.37 (0.09–1.65)	0.18
History of intestinal surgery (yes vs. no)	0.95 (0.10–8.79)	0.97	Paresthesia	0.99 (0.98–1.01)	0.62
History of abdominal imaging (yes vs. no)	0.91 (0.34–2.42)	0.84	Hearing or visionary impairments	0.98 (0.96–1.00)	0.39
History of abdominal sonography (yes vs. no)	1.02 (0.44–2.36)	0.97	Muscle pain	0.22 (0.04–1.15)	0.05
**Personal habits (yes vs. no)**			Bone pain	0.95 (0.19–4.68)	0.95
Soil playing	0.94 (0.39–2.26)	0.89	Acute onset of abdominal pain	0.95 (0.92–0.99)	0.18
Special diet	0.76 (0.23–2.48)	0.65	Chronic abdominal pain	0.89 (0.42–1.88)	0.77
Frequent use of spices or medicinal plants	1.46 (0.17–12.51)	0.73	Recurrent abdominal pain	1.35 (0.64–2.85)	0.42
Pika	0.224 (0.043–1.62)	0.053	**Family issues (Yes vs. no)**		
			Mothers’ employment status	0.95 (0.26–3.53)	0.94
Ice eating	0.38 (0.09–1.67)	0.18	Mothers’ educational status (school diploma and above)	1.97 (0.92–4.23)	0.07
Paint wall eating			Mothers’ educational status (school diploma and above)	1.97 (0.92–4.23)	0.07
Toy paint eating	0.99 (0.98–1.01)	0.62			
Playing with toys made of plastic	0.51 (0.24–1.09)	0.07			
			Fathers’ educational status (14-year schooling and above)	1.57 (0.70–3.53)	0.27
Playing with toys made of metals	1.33 (0.41–4.34)	0.64	Fathers’ educational status (14-year schooling and above)	1.57 (0.70–3.53)	0.27
Playing with toys made of cotton	1.22 (0.37–3.96)	0.74			
Playing with toys made of plastic and cotton	2.80 (1.07–7.29)	0.03	Positive history for parents’ addiction habit (yes vs. no)	2.57 (0.71–9.31)	0.14
Playing with toys made of plastic and metal	1.43 (0.37–5.60)	0.60			
Playing with toys made of plastic	0.51 (0.24–1.09)	0.07			

ROC curve test found the age of five years as the best cutoff point with the highest sensitivity and specificity. However, the logistic regression model highlighted the educational level of the mothers as a single prominent factor with a significant difference between the two groups of the study (Table 3).

**Table 3:** Logistic regression analysis for predicting blood lead level more than 5 mic/dL

Dependent variable	Independent variable	Beta	SE of beta	OR (95% CI)	Model Significance and Nagelkerke R square
BLL ≥ 5 µg/dL	Educational status of the mothers (more or equal than 14 years schooling) (yes vs. no)	0.926	0.469	2.5 (1.0, 6.3)	0.013 and 0.239

*P* values which were below 0.2 including “Playing with toys made out of plastic “Playing with toys made out of plastic and cotton”,” Playing with toys made out of plastic”, “Positive history for parents’ addiction habit”, “Mothers’ educational status”, “Ice eating (yes vs. no)”, “PIKA”, “muscle pain”, “Acute onset of abdominal pain “, “Pain in extremities”, “Pain in extremities”, “performing endoscopy”, “Wall sleek oil colored”, “Living near to industrial zones”, “age”, “sex”, “weight”, and “height” were entered in logistic regression as can be found below

This factor even showed a significant difference in a higher level of BLL (BLL ≥ 10 µg/dL; *p* = 0.007, OR 1.087, CI 95% 1.026–1.152). All eight patients with BLLs ≥ 10 µg/dL were mothers with more than 14 years schooling.

There were no cases of paint consumption, or other types of eating disorders (rather than what is mentioned) in the patients.

## Discussion

This descriptive study was conducted to find the prevalence and contributing factors to elevated BLL among the pediatric patients presenting to two pediatric centers with abdominal pain. Among factors, age, weight, and height were significantly different between those with normal and elevated BLL.

Elevated BLL was more common in children younger than five years. This is felt to be mainly due to the children's behaviors. At this age range, hand mouth behaviors are more prominent in comparison to older children. This behavior makes lead exposure through the mucosal surfaces more probable [15].

Moreover, the current study found a significant difference in the BLL of the children who played with cotton and plastic toys. The presence of lead in low-quality plastic toys and imported toys has been always a concern. Interestingly, in the current study among, plastic toys, metal toys, cotton toys, and plastic cotton toys, only the last showed a significant connection to elevated BLL. This could be because these toys may contain lead or be contaminated with dust and pollution. This issue as well as the expression of hand mouth behaviors especially in younger children may be responsible for the elevated BLL.

Lead exposure remains a major environmental health concern. Although several measures have been legislated and conducted to eradicate environmental lead poisoning, it still occurs due to various sources including lead-contaminated candies and their wrapper [16, 17], toys [18], lead-based paints in old buildings [19], lead-polluted water [20], and air pollution [7]. The health impact of pediatric lead poisoning over the life of a child may be significant as it may interfere with the proper neural development of the children affecting their cognition, emotions, and behaviors. The US CDC has introduced 5 µg/dL as the threshold for interventions; however, there some evidence that even lower BLLs may exert deleterious effects on the central nervous system.

Human lead kinetics are classically (and over-simplistically) referred to as a three compartments model. These pools have an interdependence and clinicians take advantage of the metal moving between compartments with the use of chelation medications. Blood is the most labile pool with a half-life of approximately 35 days. Soft tissue is the second compartment which has a half-life of approximately 40 days [21]. The most stable compartment is in bone where lead is taken up instead of calcium. This compartment is very stable with a half-life measured in years [22]. Currently, some believe there are 20 compartments [23]. The multi-compartment models that have been proposed over the years are analyzed in detail by the US CDC [24]. Recurrent lead poisoning episodes can develop and cumulative adverse effect on the central nervous system may occur. Additionally, it has been demonstrated that lead can pass the placenta affecting the neural development of the fetus [25]. This fact makes children and pregnant women the most susceptible groups in lead toxicity.

Although the above-mentioned factors were significantly different between the groups in the univariate analysis, only the education level of the mothers was significant by logistic regression. Interestingly, children of the mothers with at least 14-year schooling certificates and university degrees had higher BLLs than non-college educated mothers. Although in the current study employment status of the mothers showed no connection to their education level, it has been suggested that it is more probable that women with higher education levels get better employments [26]. Therefore, it could be hypothesized that educated mothers in metropolises of Iran spent more time out of the home. This will predispose the lead-contaminated dust and air particles containing lead to be carried into the home environment by the mothers who are in close contact with their children. Also, it can be concluded that housekeeping mothers are more engaged in the cleaning activity in comparison to employed mothers. By removing dust, a major source of lead would be eradicated.

In addition, this study found that districts 7, 12, and 17 are the districts with the highest odds of lead poisoning. This issue is another clue on the possible role of traffic related air pollution. Tehran has a base population of 8.7 million people that increased to approximately 14 million during daytime [27]. This increases vehicle exhaust and dust since these districts are in the south and central part of Tehran which has the highest traffic flows.

In the current study, 36 patients (19.3%) had BLL ≥ 5 µgr/dL. Ataee et al. have reported that nearly half of the pediatric patients who were referred to them with abdominal pain and constipation had elevated BLL while no one in the control group had BLL ≥ 5 µgr/dL [28]. Maleknejad and colleagues conducted a study on the prevalence of elevated BLL among pediatric patients in the northern provinces of Iran. They reported that almost 38% of the patients had elevated BLL [29]. These odds underline the importance of GI symptoms and the need for the consideration of lead poisoning as a differential diagnosis.

In the study conducted by Hatami and assistants, BLL was checked in 7-year-old children population in Bushehr province of Iran in the first grade of the primary school. About 36% had BLL ≥ 5 µgr/dL. The interesting fact is that Bushehr province is one of the country zones with a low level of lead pollution [30]. In another study, Hosein Zadeh and coworkers evaluated the prevalence of elevated BLL among 144 random elementary students in one of the most polluted districts of Tehran. They reported that nearly 32% of the cases had BLL ≥ 5 µgr/dL which shows a slightly higher risk compared to the current study [31]. This rate is far higher than that observed among US children immigrants which is almost 20% [32]. This highlights the need for implantation of screening tools for BLL assessment and probable interventions. Evaluation of the pregnant mothers in their first trimester of pregnancy and the children after birth in annual intervals until age six provides appropriate care for these groups. It can also help build a national map identifying the areas with the highest level of lead contamination [33].

Specific attention should be paid toward the populations with lower socioeconomic status. People with low socioeconomic status may live in areas with higher lead contamination, for example in older buildings with old metal pipes and paints containing lead and they may not have the budget for the eradication of lead sources in their living environment. Also, they may not afford to seek medical care when the symptoms emerge.

## Limitations

One of the major limitations of the current study is the lack of a national map on the lead pollution throughout the country. Also, no precise data is available about the severity of lead pollution among various districts of Tehran. Restriction of evaluation to two centers, and small sample size makes it difficult to evaluate an exact comparison of elevated BLL among patients from different provinces of Iran and Tehran. In addition, this study has focused on the BLL of the patients with abdominal pain and no measurement for parents to see if they are exposed to lead. Presence of a pediatric group without the abdominal pain would help making a better comparison and will be considered for the future studies.

## Conclusions

This study is a reminder that lead poisoning is still a cause for pediatric abdominal pain. Age was a contributing factor to higher BLL and children less than 5 years old were more vulnerable to high BLL. However, we cannot identify causality based on this study, and exposures are multifactorial. Educated mothers may have children with high BLL. The mechanism should be elucidated if this is related to cleaning the home or it is transfer of outdoor dusts containing lead.

## Data Availability

The data generated by and used in the study is available from the corresponding author upon reasonable request.

## Abbreviations

BLL: Blood lead level
CBC: Complete blood count
CDC: Centers for Disease Control and Prevention
CI: Confidence interval
GI: Gastrointestinal
IQ: Intelligence quotient
OR: Odds ratio
